# Unresolved issues and controversies surrounding the management of colorectal cancer liver metastasis

**DOI:** 10.1186/s12957-014-0420-6

**Published:** 2015-02-18

**Authors:** Woubet T Kassahun

**Affiliations:** Department of Surgery II, Faculty of Medicine, Clinic for Visceral, Transplantation, Thoracic and Vascular Surgery, OKL, University of Leipzig, Liebig Str. 20, 04103 Leipzig, Germany

**Keywords:** Colorectal cancer, Colorectal cancer liver metastases, Liver resection, Timing of liver resection

## Abstract

Ideally, tumors that might cause morbidity and mortality should be treated, preferably early, with proven, convincing, and effective therapy to prevent tumor progression or recurrence, while maintaining a favorable risk-benefit profile for the individual patient. For patients with colorectal cancer (CRC), this diagnostic, prognostic, and therapeutic precision is currently impossible. Despite significant improvements in diagnostic procedures, a sizable number of patients with CRC have liver metastases either at presentation or will subsequently develop it. And in many parts of the world, most cancer-related deaths are still due to metastases that are resistant to conventional therapy. Metastases to the liver occur in more than 50% of patients with CRC and represent the major determinant of outcome following curative treatment of the primary tumor. Liver resection offers the best chance of cure for metastases confined to the liver. However, due to a paucity of randomized controlled trials, its timing is controversial and a hotly debated topic. This article reviews some of the main controversies surrounding the surgical management of colorectal cancer liver metastases (CRLM).

**Electronic supplementary material:**

The online version of this article (doi:10.1186/s12957-014-0420-6) contains supplementary material, which is available to authorized users.

## Introduction

Despite significant improvements in diagnostic procedures, surgical techniques, local and systemic treatment options, and general patient care, most cancer-related deaths are still due to metastases that are resistant to conventional therapies. Next to lymph nodes, the liver is the most common site of metastases from human solid tumors [1]. Metastases to the liver occur in more than 50% of patients with CRC and represent the major determinant of outcome following curative treatment of the primary tumor [2]. Indeed, for nearly one-third of patients with CRC the liver is the only site of metastatic disease [1]. About 14% to 35% of patients with CRC have liver metastases at presentation, and another one-third will subsequently develop it [3-8]. Liver resection offers the only chance of cure for metastases confined to the liver with 5-year overall survival rates up to 58% [9-12]. Unfortunately, the majority of patients with CRLM are not amenable to surgery initially, and the reported rate of successful resection has ranged from 20% to 30% [13,14]. Some of the reasons for the low rate of successful liver resection are: disseminated disease with concomitant extrahepatic metastases, lack of a sufficient future remnant liver, and unfavorable anatomical location in the liver. These patients may become surgical candidates following conversion chemotherapy that downsize metastatic disease [15]. In appropriately selected patients with primarily inoperable liver metastases, secondary resection after downstaging chemotherapy may afford long-term outcomes comparable to primary resection [15,16]. Overall, however, despite the advances made in the last decades, outcome after liver resection for CRLM as a primary or a two-stage resection is heterogeneous, and many patients still do poorly even after careful selection and a successful removal of the metastatic lesion [17]. The main problem is that patients who actually undergo successful liver resection have a high risk of developing recurrent disease, either in the liver or elsewhere. This occurs in approximately 60% to 70% of patients after primary liver resection and roughly one-third of these patients die within 2 years of surgery for CRLM [18-20]. Patients with untreated liver metastases have a median survival of less than 12 months and a 5-year survival rate of almost zero [21-23]. Due to lack of convincing evidence, there are areas of controversies related to treatment protocols of patients with CRLM. Almost all studies regarding these controversial issues are nonrandomized studies with inherent limitations and representative data from prospective randomized trials is still missing. In this contribution, I focus on current controversies surrounding the management of CRLM (Table 1).

**Table 1:** **Summarized highlights of controversies surrounding the management of colorectal cancer liver metastases**

**Treatment strategy**	**Arguments in support of**	**Arguments against**
The simultaneous approach	No increase of morbidity and/or mortality in carefully selected patients	Considerable increase of morbidity and/or mortality
	Removal of all cancer in a single procedure; thereby lowering the risk of disease dissemination	No time-test approach to evaluate the biological behavior of metastasis and this may result in unnecessary liver resection in rapidly progressing disease
	Similar PFS and OS compared to those with staged resection	Higher recurrence rate and a negative impact on long-term outcome
Pre-HR chemotherapy	Decreases the magnitude of resection	Delays liver resection and may result in a unresectable state in nonresponders
	Eradicates micrometastases	May lead to liver parenchyma damage and increased postoperative morbidity
	Increases R0 resection rates	No impact on PFS and OS
	Assesses responsiveness to specific chemotherapy, thus, identifying and selecting patients with favorable tumor biology. It improves PFS	
Extensive resection for DLM	Response on imaging does not necessarily signify clinical or pathological response ( in up to 83% evidence of residual disease); so resect all initial sites if possible, despite disappearance on imaging	Hence, durable clinical response is as high as 62%, resect only residual macroscopic disease leaving the disappeared lesions *in situ* or alternatively, continue systemic chemotherapy alone
The liver-first approach	It is the liver metastasis, rather than the primary tumor, that gives rise to systematic metastatic disease, so it should be addressed first	No, it is the primary tumor that produces systemic effects promoting angiogenesis in the liver, thus favoring the spread of metastatic disease
	It avoids the risk for progression of CRLM while the patient is treated for the primary tumor, especially if complications are encountered; thereby improving median survival and 3-year survival rates	Despite apparently similar treatment protocols in those few studies, the variations in survival rates of the liver-first approach are wide; so its comparison with the bowel-first approach or the combined strategy is problematic
	Option to give systemic chemotherapy as a first step early in the treatment course that may lead to an effective response in the primary tumor and avoids resection	

CRLM, colorectal liver metastasis; DLM, disappearing (no longer visible on imaging) liver metastases; HR, hepatic resection; PFS, progression free survival; OS, overall survival; Pre-HR chemotherapy, neoadjuvant chemotherapy for resectable CRLM.

## Review

### Simultaneous versus staged resection of colorectal cancer liver metastases

One of the controversial issues is the optimal timing of liver resection for resectable synchronous colorectal liver metastases (SCRLM). Traditionally, colorectal cancer and SCRLM have been approached with staged initial resection of the primary tumor followed by liver resection. But the paradigm for surgical management of SCRLM has begun to change, with authors reporting good results for simultaneous surgical removal of the primary tumor and the liver metastases [24-26]. In the simultaneous approach, the liver metastases and the primary tumor are resected at the same time. The main objective of this approach is cure thorough the removal of all cancer tissue during a single operation, thus, avoiding delay of surgical treatment of liver metastases. Some evidence suggests that patients with SCRLM who are treated with simultaneous resection of CRC and CRLM have similar long-term outcomes to those treated with a staged approach; and the simultaneous approach does not increase the morbidity or mortality associated with liver resection [27-33]. The major limitation of the simultaneous approach is that the mortality and morbidity of major liver resection, combined with bowel resection, are likely to be considerable; in some studies as high as 8% and 36% respectively [34-37]. In a large retrospective review, Reddy *et al*. [37] found that simultaneous colorectal and major liver resections increased morbidity and mortality by more than threefold compared with major liver resection alone and that the increased severe morbidity was maintained when compared with the added severe morbidity of staged colorectal plus liver resection. According to these authors, simultaneous procedures should only be pursued for minor liver resections with application to major liver resection on a selected basis. Furthermore, in agreement with others [38], these authors recommend avoiding the simultaneous approach in the following situations: 1) when SCRLM is noted on exploration for emergency situations such as colonic perforation (high risk of occult distant metastatic disease), bowel obstruction and bleeding; 2) in those patients with an extremely increased risk of postoperative liver failure, such as patients with liver cirrhosis or other long-standing chronic liver disease; and 3) in patients in whom the future remnant liver will provide inadequate hepatic function. A meta-analysis of 2,880 patients with SCRLM who were followed up for at least 36 months reported that simultaneous resection is as safe as delayed resection as long as patients were less than 70 years old and without severe coexisting disease [39]. Similarly, in the study of Thelen *et al*. [40], patients with the simultaneous approach had far higher mortality than staged, which was associated with age >70 years and major hepatectomy. Furthermore, a time test approach has been suggested by some authors favoring the staged strategy to evaluate the biological behavior of the metastatic disease, to treat potentially occult disease, and to avoid liver resection in patients with rapidly progressing disease [41]. And lastly, there is some evidence that the simultaneous approach has a negative effect on progression-free survival (PFS) [37,38,42]. Therefore, given the results of the above-mentioned studies (all nonrandomized retrospective) and other results [25,26,34,43] (Table 2), the simultaneous approach can be offered only in a highly selected subset of patients pending additional high-quality evidence. Currently, no randomized controlled trials on the relative merits of different therapeutic approaches are available; thus, there is no evidenced surgical practice that became as standard of care for resectable SCRLM. Thus, in evaluations of the utility of simultaneous resection of CRC and CRLM, surgeons should carefully consider the additional morbidity and mortality associated with the procedure, as well as the possible improvement in survival.

**Table 2:** **Large retrospective studies focusing on comparison of the simultaneous versus the staged approach for the treatment of colorectal cancer liver metastases**

**Author**	**N** ^**a**^	**Year**	**Approach, n**	**Morbidity, %**	**Mortality, %**	**Conclusions**
Capussotti [29]	79	2007	31 simul	33	0	Mortality rates are similar in both procedures, so the simultaneous procedure can be performed in carefully selected patients
			48 staged	56	1,3	
Lyass [25]	112	2001	26 simul	27	0	Because of lower mortality rates and similar OS compared to staged, Simultaneous resection is a safe and efficient procedure for the treatment of resectable SCRLM
			86 staged	35	2.3	
Bolton [35]	165	2000	50 simul^b^	nr	17	The mortality rate is higher if liver resection is combined with colorectal resection. Therefore, patients should have hepatic resection delayed for at least 3 months after colon resection
			115 staged	nr	1	
de Haas [42]	228	2010	55 simul	11	0	The simultaneous approach is safe for limited HR
			173 staged	25	0.6	However, the higher recurrence rate observed in studied patients makes its oncological value and use in clinical practice questionable
Martin RC [33]	230	2009	70 simul	56	0	Morbidity and mortality rates are comparable in both procedures. Therefore, Simultaneous resection is an acceptable option in patients with resectable SCRLM
			160 staged	55	4	
Martin R [26]	240	2003	134 simul	49	2	Simultaneous resection should be considered a safe option in patients with resectable SCRLM, because it offers reduced morbidity, shorter treatment time, and similar survival outcomes
			106 staged	67	2	
Reddy [37]	610	2007	135 simul	36	1	Simultaneous resection is safe and should be considered for patients with SCRLM; however, due to higher morbidity compared to staged resection only in those patients whose hepatic tumor burden is amenable to minor liver resection (less than three segments)
			475 staged	18	0.5	
Nordlinger [34]	1008	1996	115 simul	nr	7	The mortality rate is increased when a major liver resection is performed simultaneously with the resection of the primary tumor
			893 staged	nr	2	
						Therefore, this procedure is recommended only if it can be done with a minor liver resection and through the same abdominal incision

^a^Only those studies with N ≥50 were considered in this table.

^b^Liver resection was carried out simultaneously with or within 3 months of colorectal resection.

HR, hepatic resection; N, total number of patients; n, number of patients treated with simultaneous or staged resection; nr, not reported; SCRLM, simultaneous colorectal liver metastasis; simul, simultaneous resection of the primary tumor and SCRLM.

### The role of chemotherapy in neoadjuvant setting

In cases of unresectable CRLM, there is no controversy as the only practical option is systemic chemotherapy. However, whether preoperative chemotherapy in patients with resectable CRLM confers a benefit in terms of decreased recurrence or improved survival is not clear. Thus, the balance between the risks and benefits of preoperative chemotherapy is a hotly debated topic [44]. Some of these debates originated decades ago, yet are still ongoing despite interim advancements in other domains of oncology. The arguments in support of preoperative chemotherapy are based on the fact that this treatment modality decreases the magnitude of resection, eradicates micrometastases, increases R0 resection rates, and aids in the selection of adjuvant therapy based on assessments of responsiveness to a specific chemotherapy. It also identifies patients with aggressive tumor biology in whom liver resection may not be appropriate. In their retrospective study of 131 patients who underwent liver resection for CRLM, Adam *et al*. [45] found that patients whose metastatic lesions progressed during chemotherapy had a significantly poorer long-term prognosis (8% versus 37% 5-year survival). However, it can be equally argued that neoadjuvant chemotherapy (NC) delays liver resection and may increase the risk of tumor progression to an unresectable state if the patient does not respond to chemotherapy [8,46-49]. Moreover, in their series of 1,471 patients with resectable metachronous CRLM, Adam *et al*. [50] compared those who received NC with those treated by liver resection alone. The authors found that postoperative complications were significantly higher in the chemotherapy group, and more importantly, NC did not improve overall survival (OS). Thus, although NC may provide earlier control of disseminated disease, it induces histologic changes in the liver including steatohepatitis and vascular injury that may result in increased postoperative complications [50-55]. Others disagree with this view [56,57]. In a randomized controlled trial of 364 patients (European Organization for Research and Treatment of Cancer: EORTC 40983) with resectable CRLM, Nordlinger *et al*. [57] compared patients who received 12 cycles of FOLFOX (6 cycles before surgery and 6 cycles after) with patients treated by liver resection alone. Their result have not shown these harmful effects and showed a progression-free survival (PFS) of 36.2% at 3 years in the chemotherapy group compared to 28.1% (*P* = 0.041) in the surgery-alone group. However, at a median follow-up of 8.5 years, no significant improvement in OS had been observed in the chemotherapy group compared to surgery alone [58]. Moreover, this study failed to prove whether the indicated benefit of PFS in the chemotherapy group related to neoadjuvant treatment, adjuvant treatment or a combination of both. In the series of Nanji *et al*. [59], a total of 284 out of 293 patients with resectable CRLM underwent liver resection without NC. These authors reported a 3-year PFS and a 5-year OS of 46.2% and 55%, respectively. These conflicting data have led many experts to dispute the necessity of administering preoperative chemotherapy for primarily resectable liver metastases altogether.

Given the benefit of PFS seen in the EORTC 40983, Nordlinger *et al*. [57,58] advocated the use of preoperative chemotherapy as the standard of care for patients with operable liver metastases. Conversely, others suggest surgery without preoperative chemotherapy for this population of patients, basing their argument on chemotherapy-induced liver injury with increased morbidity, the potential loss of that particular regimen of chemotherapy from future lines of treatment, and the nonbeneficial effect of chemotherapy on long-term survival [59-61] (Table 3).

**Table 3:** **Review of large studies** (**all retrospective except** [57,58]) **focusing on a comparison of neoadjuvant chemotherapy followed by hepatic resection versus hepatic resection alone for resectable colorectal cancer liver metastases**

**Author**	**Year**	**N** ^**a**^	**TT, n**	**Morbidity, %**	**Mortality, %**	**PFS, mo**	**OS, m**	**Conclusions**
Mehta [54]	2008	173	130 NC+HR 43 HR alone	nr	nr	nr	nr	NC was associated with regimen-specific hepatic injury. However, this did not increase postoperative morbidity and Mortality
				nr	nr	nr	nr	
Scoggins [55]	2009	186	112 NC+HR	49	0	40	56	Similar remarks as Mehta et. Al
			74 HR alone	47	7	56	65	
Pawlik [53]	2007	212	153 NC+HR 59 HR alone	35	0	nr	nr	Preoperative chemotherapy is associated with hepatic injury in 20 to 30% of patients and the type of injury was regimen-specific
				30	4	nr	nr	
Nordlinger [57,58]	2008	364	151 NC+HR 152 HR alone	25	0.66	19	61	In resectable CRLM, ^b^Chemotherapy does not improve OS. However, it improves PFS
				16	1.3	12	54	
Vauthey [52]	2006	406	248 NC+HR 158 HR alone	23	14.7	nr	nr	NC induces regimen-specific significant liver injury and increases mortality after liver resection
				18	1.6	nr	nr	
Welsh [56]	2007	497	252 NC+HR	29	2	nr	nr	Liver resection for CRLM is safe following NC
			245 HR	27	2	nr	nr	
Reddy [61]	2009	499	297 NC+HR	nr	nr	nr	53	NC was not associated with improved RFS and OS
			202 HR alone	nr	nr	nr	36	
^c^Adam [50]	2010	1471	169 NC+HR	37	2.1	nr	nr	NC did not improve the outcome of patients with resectable CRLM
			1302 HR alone	24	1.9	nr	nr	

^a^Only those studies with N ≥150 and relatively comparable number of patients in both treatment options were considered in this table.

^b^Chemotherapy was administered as perioperative (before and after hepatic resection); ^c^PFS and OS are reported not in months but in % and there is no significant difference in both groups.

HR, hepatic resection; N, total number of patients; n, number of patients in either of the treatment options; NC, neoadjuvant chemotherapy for liver metastases; nr, not reported; TT, type of treatment.

In summary, the final decision on the timing of liver resection (upfront or after NC) should depend on the preference of the patient and of the interdisciplinary tumor board that includes the surgeon, the radiologist, the oncologist, the radiation therapist, and others working together. It should be made for the individual patient on the basis of the best available medical evidence in the context of the clinical situation.

Regarding adjuvant chemotherapy (AC) after liver resection, there are several studies that have demonstrated its benefit [19,62-66]. These positive results were taken by many as an answer to the benefit of AC after liver resection, and its use has become routine. However, the majority of these studies are retrospective. No randomized controlled trials have examined the benefit of AC after curative liver resection; any such trial would require a large sample size and prolonged follow-up. Two prospective randomized trials that intended to show the benefit of AC had to close prematurely because of slow accrual, thus lacking the statistical power to demonstrate the predefined difference in survival [67,68]. Therefore, the benefit of AC after liver resection for CRLM has not been rigorously validated in clinical trials.

### Disappearing (no longer visible on imaging) liver metastases

Given recent therapeutic advances, the nihilism that decades ago often characterized the treatment of patients with CRLM has been replaced by a measure of excitement. Along with advances in operative technique and imaging that have clearly contributed to improvements in the management of patients with CRLM, recent therapeutic advances have been stimulated in part through identification of cellular processes characteristic of CRC that permit therapeutic targeting with favorable therapeutic index. Following the introduction of new, more effective cytotoxic agents, tumor response has improved significantly [69-74].

Disappearing liver metastases (DLM) refers to the complete response or disappearance of a liver metastasis on imaging after administration of preoperative chemotherapy [75]. Depending on the quality and type of cross-sectional imaging, DLM occur in up to a quarter of patients who undergo preoperative systemic chemotherapy either in neoadjuvant setting for initially resectable liver metastases or as conversion chemotherapy for initially unresectable metastases in an attempt to bring patients to potentially curative resection [75-79]. Small size of the metastatic lesion (less than 2 cm) and prolonged duration of systemic chemotherapy may increase the likelihood of DLM [78,79]. The management of DLM is challenging because a complete response on imaging does not necessarily signify a complete clinical or pathological response. It can be the result of chemotherapy associated parenchymal changes to the liver such as steatosis and steatohepatitis that alter the imaging characteristics of the liver and lead to a reduction in the sensitivity of imaging during chemotherapy [80,81]. A durable clinical response for DLM occurs in about 20% to 50% of patients treated with systemic chemotherapy alone, leaving a subset of patients with undiagnosed microscopic liver metastases, which develop subsequent intrahepatic recurrence [75,79]. Study results regarding the outcome of DLM are discrepant and conflicting. In a report of Elias *et al*. [82], 27% of the DLMs were identified at laparotomy, whereas recurrence occurred in 20% of the studied patients. In the remaining 53%, follow-up showed no recurrence after 31 months. In their second series Elias *et al*. [77] reported that 10 (62%) out of 16 patients with DLM remained recurrence-free at 51 months. In the series of Auer *et al*. [78], 17 (44%) out of 39 patients with DLM developed an intrahepatic recurrence, 8 (21%) patients developed a recurrence at an extrahepatic site, and 17 (44%) patients remained recurrence-free at a median follow-up of 40 months. These authors found that the use of hepatic arterial chemotherapy, disappearance of the metastatic lesion on MRI and normalization of serum carcinoembryonic antigen (CEA) as independent predictors of a true response. By contrast, in the study of Benoist *et al*. [76] macroscopic residual disease was found in more than 25% of DLM during laparotomy at the site of liver metastases that were considered to have disappeared on imaging. Overall, these authors observed evidence of residual disease in 83% of DLMs.

To summarize, although the beneficial effect of resection of hepatic metastases on survival has been clear, the extent of primary surgery needed in DLM has not. Thus, controversy persists with regard to the extent of surgery required for patients with DLM. There are several proposed management strategies for this subset of patients. These include: resection of all initial sites of DLM when possible; surgical removal of residual macroscopic disease while leaving the disappeared lesions *in situ* if the resection would be too extensive, leaving insufficient remnant liver; resection followed by additional adjuvant chemotherapy; continuing systemic chemotherapy alone; and others. No strong evidence from randomized controlled trials exists to support any of these management options, particularly the routine use of risky and extensive liver resection in patients with DLM. For now, clinicians must use their best judgment taking a risk-benefit approach to establish the extent of surgical treatment. In the future, decision aids, such as one tested in a randomized controlled trial, might play a greater part in decision making about the treatment strategies of DLM.

### The liver-first approach

Contrary to the classic bowel-first approach, the liver-first approach is the reverse of the classic approach and begins with systemic chemotherapy directed against the liver metastases, followed by liver resection, then chemoradiotherapy for patients with rectal cancer, and colorectal resection as the last surgical step. This approach has been proposed for patients with advanced synchronous CRLM, and in particular for those whom the primary is located in the rectum and is asymptomatic [83]. In relation to chemotherapy, treatment after liver resection could be regarded as neoadjuvant for the primary disease, and adjuvant treatment is given after surgery for both liver metastases and colorectal cancer. Given the prognostic decisive role of liver metastases in long-term survival, the liver-first approach has a theoretical advantage. This new trend has been driven in part by efforts to improve survival. The option of giving systemic chemotherapy early in the treatment course provides a chance to evaluate a response and thereby define the tumor biology of the metastatic lesions. In addition the liver-first approach is the result of the assumption that it is the liver metastasis, rather than the primary tumor, that gives rise to systemic metastatic disease. It underlies the importance of prioritizing treatment of the most problematic component of the patient’s disease. Thus, considering the time needed for neoadjuvant chemoradiation and resection of rectal cancer, which is longer than 3 months [84], addressing the liver first may avoid the risk for progression of CRLM while the patient is being treated for the primary tumor, which is a concern in the classic approach. This view has been supported by some studies (all nonrandomized) that showed a median survival up to 44 months and 3-year survival rates as high as 83% [8,46-48]. Data to support this argument is limited, and there is a counterevidence to suggest that it is the primary tumor that produces systemic effects promoting angiogenesis in the liver and thus favoring the spread of metastatic disease [85,86]. Moreover, despite apparently similar treatment protocols used in those few studies, the variations in survival rates of the liver-first approach are wide [47,48]. This makes its comparison with other treatment protocols, such as the bowel-first approach or the combined strategy, difficult. An adequately powered randomized controlled trial examining the effect of the liver-first approach on recurrence and long-term survival might be logistically challenging, because of the need for a large sample size. Thus, to date, no randomized, controlled studies have assessed the benefits of this modern strategy or its effects on recurrence and long-term survival of patients with CRLM.

### Unresectable liver metastases and asymptomatic primary tumor

Historically, the common treatment strategy in patients with asymptomatic primary tumor with synchronous unresectable liver metastases is surgical removal of the primary tumor followed by systemic chemotherapy. The theoretical basis that supports this approach is that the growth of the primary tumor is the main cause of major complications such as intestinal obstruction, perforation and hemorrhage, so resection should be more likely to prevent these complications while achieving best palliation in terms of quality of life [87-89]. Further, resection of the primary tumor reduces the risk of the growth of additional metastatic disease that may influence long-term outcomes. Results of retrospective studies show that with an estimated survival advantage of 6 months, primarily resected patients live significantly longer than non-resected patients [90-93]. Moreover, patients put on initial systemic chemotherapy tended to have more major complications that mandated surgical intervention and termination of chemotherapy. Bowel obstruction is the most frequently encountered complication during chemotherapy with a rate ranging from 5.6% to 29% followed by bleeding from the primary tumor that was experienced by 3% to 5% of the patients [90,93-95].

On the other side, results of comparative studies demonstrate that most patients with widely metastatic disease will not experience a complication resulting from an non-resected primary tumor, and that the rate of intestinal obstruction due to subsequent growth of the primary tumor is similar to that caused by adhesions after colorectal resection [96]. Initial systemic therapy is safe and patients put on this treatment modality showed an overall response rate as high as 70% [18,92,97]. According to one report, 93% of patients initially treated by systemic chemotherapy never required emergency surgery [98]. Overall morbidity and mortality after resection for advanced metastatic colorectal cancer were as high as 21% and 16%, respectively [99,100]. This figure is significantly higher than that of colorectal cancer in general [100,101]. Moreover, experimental data show that resection of the primary tumor may enhance the growth and proliferation of metastatic cells [89,102,103]. The underlying mechanism is thought to be lack of a circulating anti-angiogenic factor, normally produced by the primary tumor that leads to suppression of angiogenesis and enhanced apoptosis and hence inhibiting metastatic progression. All this suggests that any benefit to preventing the growth of additional metastases by resection of the primary tumor does not influence long-term outcome.

In summary, studies examining the effect of primary surgery on long-term outcomes in patients with asymptomatic CRC and unresectable CRLM have limitations, and the routine use of surgery in all patients in this group might not be justified. Therefore, surgery is no longer routinely considered as an initial therapeutic intervention, and the appropriateness of resection has been questioned in this setting. Thus, recently resection is hesitantly offered as an alternative with ill-defined indications and mixed results.

## Conclusions

The classic, combined, and reverse strategies are associated with similar outcomes. While some authors argue that the mortality and morbidity of major liver resection, combined with bowel resection, are likely to be considerable, with some studies showing an increase of more than threefold, others disagree with this view. Despite experiences reported by some authors, there is no consensus as yet for the role of the liver-first strategy in improving long-term survival in patients with CRLM. The benefit of chemotherapy in neoadjuvant or adjuvant setting for resectable CRLM has not been rigorously validated in clinical trials. There are several proposed management strategies for patients with disappearing liver metastases. However, no strong evidence from randomized controlled trials exists to support either of these management options.

**Figure 1:**
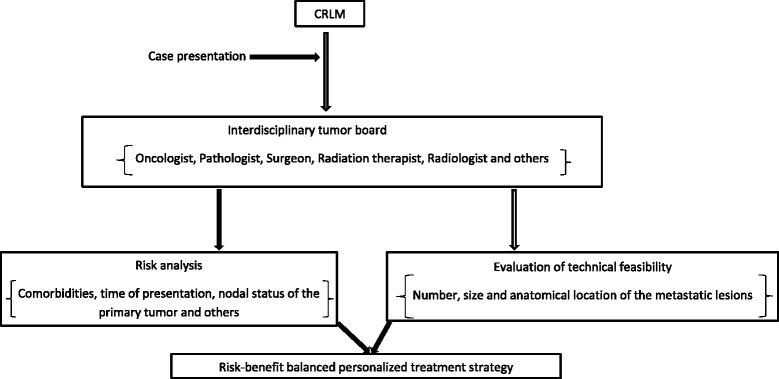
**Summary of general management strategy for patients with colorectal cancer hepatic metastases.** CRLM, colorectal cancer liver metastases.

All these controversies surrounding the management of CRLM underscore the need for large-scale, multicenter randomized trials to better define effective therapeutic strategies for clinically meaningful long-term outcomes. In the absence of high-quality evidence, disease management of CRLM needs to be personalized, balancing potential risks and benefits of treatment (and related uncertainties) with risk of the disease (Figure 1).

## Abbreviations

AC: Adjuvant chemotherapy
CRC: Colorectal cancer
CRLM: Colorectal cancer liver metastases
DLM: Disappearing liver metastases
EORTC: European Organisation for Research and Treatment of Cancer
HR: Hepatic resection
NC: Neoadjuvant chemotherapy
OS: Overall survival
PFS: Progression-free survival
SCRLM: Synchronous colorectal liver metastases
